# Flagging fusion: Phosphatidylserine signaling in cell–cell fusion

**DOI:** 10.1016/j.jbc.2021.100411

**Published:** 2021-02-11

**Authors:** Jarred M. Whitlock, Leonid V. Chernomordik

**Affiliations:** Section on Membrane Biology, Eunice Kennedy Shriver National Institute of Child Health and Human Development, National Institutes of Health, Bethesda, Maryland, USA

**Keywords:** membrane fusion, phosphatidylserine, myogenesis, osteoclast, placenta, Ca^2+^-PLS, Ca^2+^-activated phospholipid scrambling, Cas-PLS, caspase-activated phospholipid scrambling, LPC, lysophosphatidylcholine, PLSase, phospholipid scramblase, PM, plasma membrane, PS, phosphatidylserine, STAB2, stabilin 2

## Abstract

Formations of myofibers, osteoclasts, syncytiotrophoblasts, and fertilized zygotes share a common step, cell–cell fusion. Recent years have brought about considerable progress in identifying some of the proteins involved in these and other cell-fusion processes. However, even for the best-characterized cell fusions, we still do not know the mechanisms that regulate the timing of cell-fusion events. Are they fully controlled by the expression of fusogenic proteins or do they also depend on some triggering signal that activates these proteins? The latter scenario would be analogous to the mechanisms that control the timing of exocytosis initiated by Ca^2+^ influx and virus-cell fusion initiated by low pH- or receptor interaction. Diverse cell fusions are accompanied by the nonapoptotic exposure of phosphatidylserine at the surface of fusing cells. Here we review data on the dependence of membrane remodeling in cell fusion on phosphatidylserine and phosphatidylserine-recognizing proteins and discuss the hypothesis that cell surface phosphatidylserine serves as a conserved “fuse me” signal regulating the time and place of cell-fusion processes.

Most of our cells, even those that function in tight contact with others, maintain their autonomy; however, cells in some tissues relinquish their individuality and fully join their volumes in order to accomplish a shared biological function (1, 2, 3, 4). These cell fusions are regularly observed in normal development and physiology, as well as wound healing and human disease. Mixing of cell genomes, organelles, and cytoskeletons upon complete unification of the volumes of two or more cells is one of the most dramatic rearrangements in cell physiology. Therefore, it comes as no surprise that cell-fusion processes are highly regulated and as diverse as the cell types where fusion is observed. The efforts of many have partially identified the protein machinery in some of the best-characterized cell fusions; however, the mechanisms by which these machines elicit syncytia formation remain elusive. Despite the disparate nature of the cells involved and the unique machinery utilized by each system, a reoccurring signal between fusing cells is the appearance of phosphatidylserine (PS) on the cell surface, a consequence of several multistep intracellular and extracellular signaling cascades (5).

Here we summarize recent advancements in the characterization of cell fusion and PS signaling, recognize gaps in our understanding, and propose hypotheses suggesting that PS exposure functions as a conserved regulator of diverse cell fusions. We start with a distilled recap of several cell-fusion processes and a brief summary of the current mechanistic understanding of membrane fusion and the molecular machines that facilitate it. We then focus on PS, its role in cell–cell signaling and the machines that manage its plasma membrane (PM) distribution. Finally, we discuss the link between cell fusion and PS exposure, PS-binding proteins and PS receptors. There, we consider current evidence suggesting that PS acts at the point of membrane remodeling between ready-to-fuse cells, in effect determining for cells a time to fuse. Based on the analysis of PS contributions to intercellular and viral fusion, we propose hypotheses for the mechanism by which PS regulates cell fusions.

## Cell–cell fusion

### Diversity of cell–cell fusion processes

**Figure 1:**
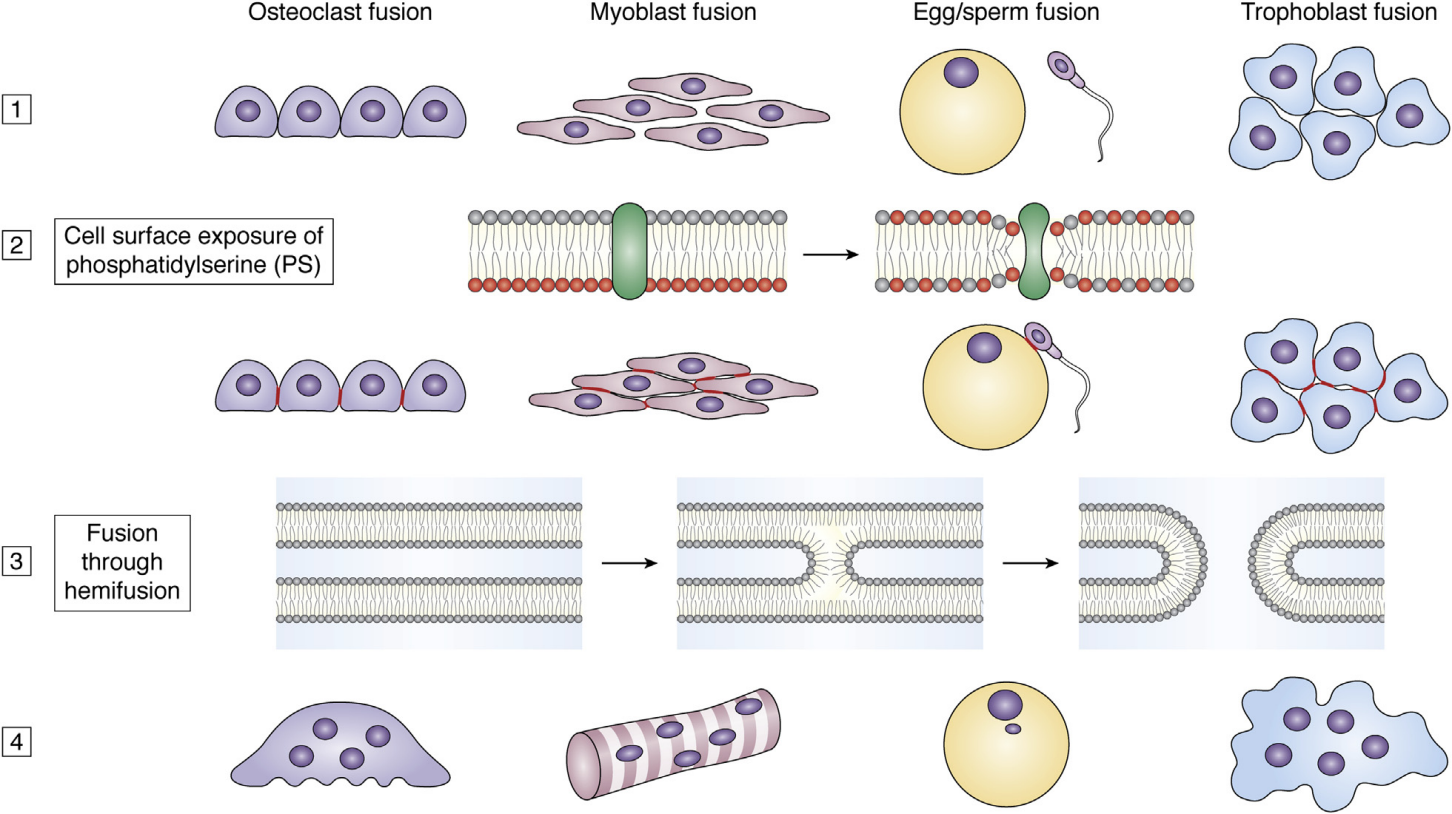
**Phosphatidylserine (PS) exposure and fusion through hemifusion unite disparate fusion processes.** Cell–cell fusion processes, including fusion of osteoclast precursors, myoblast fusion, sperm–egg fusion, and trophoblast, are preceded by dissimilar differentiation processes (1) and produce cells (osteoclasts, myotubes, zygote, syncytiotrophoblast) strikingly different in their properties and functions (4). In spite of this diversity, the actual membrane fusion event in all these processes apparently proceeds through a conserved pathway of membrane rearrangements, in which PS exposure on the cell surface (2) is followed by formation of early hemifusion intermediates and the opening of fusion pores (3).

In many tissues, cells fuse under normal physiological conditions: myoblast fusion generates skeletal muscle fibers (3); fusion between cells of the monocyte/macrophage lineage produces osteoclasts (6); cytotrophoblasts fuse into huge placental syncytiotrophoblasts at the maternal–fetal interface (7) and sperm/egg fusion yields zygotes (8) (Fig. 1). In inflammatory response, macrophage fusion into foreign-body giant cells is a hallmark of the host response to material implantation and/or some infections (9, 10). In disease, cell fusion has been implicated in the initiation and progression of cancer (11, 12, 13) and in some viral infections (14). Moreover, fusion of mesothelial cells has recently been suggested to be a major cause of postoperative and dialysis-related complications in postsurgical adhesion (15). The biological function fulfilled by fusing cells into multinucleated syncytia seems to differ between different cell types. Multinucleated skeletal muscle cells house long actin-myosin fibers capable of generating force between remote anchored positions. Large multinucleated osteoclasts are more effective in bone resorption than their precursors. The larger sizes of multinucleated giant cells and their extensive membrane ruffling help them to surround and phagocytose larger foreign particles. The massive syncytiotrophoblast layer creates a unified barrier between a mother and her fetus, while also manufacturing and dispersing essential messengers that guide embryonic development. Moreover, cell fusion sculpts the organs and defines body shape in *C. elegans* (2). While cell-fusion processes widely differ in their biological roles, they do involve several consistent intermediates.

In each cell-fusion process, progenitors of all strides must proceed through prefusion steps (differentiation-related changes in protein expression, migration, fusion partner adhesion), fusion-stage membrane rearrangements, and postfusion reorganization of the cells into a syncytium (expand fusion pores to fully join cytoplasms, rearrange shared cellular components, begin specialized syncytial functions). Despite these common mechanistic features, each cell-fusion process undergoes these steps with a distinctive flare. For instance, the cell-fusion stage of muscle regeneration is preceded by activation, proliferation, and migration of progenitor cells to sites of damage (16). These progenitors then differentiate, align, adhere to each other, and rearrange cytoskeletons at contact sites (reviewed, (16)). Bone-resorbing osteoclasts begin as mononuclear bone marrow and peripheral blood cells (17). Differentiation of these cells into mononuclear osteoclast precursors is triggered by factors released by osteoblasts and osteocytes within the bone matrix. Osteoclast precursors then align and adhere to one another (18, 19). Fusion between a syncytiotrophoblast and underlying cytotrophoblasts is less characterized, but also follows days of proliferation, differentiation, and aggregation of cytotrophoblasts (20). Each of these and other cell fusions involves multiple factors, including cytokines, hormones, protein kinases, transcription factors, proteases, adhesion proteins, and many membrane receptors long before any actual membrane remodeling takes place. After days of relatively slow and asynchronous development of fusion competence, actual fusion takes only seconds or minutes (21, 22). Of course, if the dance of prefusion misses any single step, the result is the same, no syncytium.

### Membrane rearrangements and protein machinery in cell fusion

Current thinking about mechanisms of cell–cell fusion is largely informed by our understanding of better characterized families of membrane fusion processes, namely intracellular fusion and the fusion stage of enveloped viral entry (2). At its heart, any biological membrane fusion consists of the merger of two lipid bilayers, and generally speaking, lipid bilayers do not fuse spontaneously. Bilayers formed from phosphatidylcholine (PC), the most abundant lipid in the membranes of mammalian cells (23), can maintain a 3 nm contact and not fuse for days (reviewed, (24)). This is much closer than the 10–30 nm gaps that separate PMs during cell–cell adhesion. Fusion of protein-free lipid bilayers requires special conditions (*e.g.*, specific lipid compositions and/or dehydration of the contact zone) and proceeds through hemifusion connections between the proximal monolayers of the bilayers before merger of the distal monolayers. Diverse biological fusion processes apparently also converge on a similar fusion-through-hemifusion pathway of lipid rearrangements, although the progression through these membrane fusion stages is thought to be controlled by proteins (reviewed, (25)) (Fig. 1). These proteins or protein complexes, often referred to as fusogens or fusion proteins, are thought to locally bend the two membrane bilayers, bringing them into very close contact (∼1 nm), facilitate formation of fusion intermediates (hemifusion and nascent fusion pores), and provide the forces that drive the fusion reaction from hemifusion to the more energy-intensive stages of opening and expanding fusion pores.

Identification of the proteins that directly mediate fusion requires experimental verification that a protein (or complex of proteins) is required for fusion, present at the time and place of fusion, and sufficient to fuse both biological membranes and model lipid bilayers that normally do not fuse (reviewed, (2)). For many intracellular and viral fusions, we know the identity of the protein fusogens and have detailed structures of their prefusion and postfusion conformations (*e.g.*, SNARE proteins, atlastins, dengue E protein, and HIV Env). While the last decade has brought about major progress in the identification and characterization of proteins involved in some cell-fusion processes, for most cell fusions, the identity of the proteins that fuse PMs remains to be established or confirmed (reviewed, (2)).

To understand mechanisms that regulate cell fusions, we first need to distinguish the machines that elicit fusion from those involved in prefusion processes that generate fusion-competent cells. The most widely utilized approach to distinguish these roles involves modifying the expression or activity of a protein in question and assessing whether this perturbs cell fusion but not expression of some late differentiation markers (for instance, (26, 27)). Of course, this approach assumes a highly regular differentiation process unable to accommodate perturbation and requires a set of reliable, temporal markers along the differentiation/fusion process. An alternative approach involves uncoupling these prefusion and fusion stages. For instance, myogenesis can be reversibly blocked upstream of fusion by omitting calcium from the media (28). Limiting cell contact during prefusion differentiation by plating cells at low density, followed by replating cells at a high density following differentiation, also uncouples membrane remodeling from cell-density independent, prefusion stages (29). In cell fusion mediated by HIV Env (30), myoblast fusion (21), and osteoclast fusion (22, 31), we have uncoupled fusion from prefusion stages using the hemifusion inhibitor lysophosphatidylcholine (LPC). LPC does not block prefusion processes, but because all cell fusions apparently begin with hemifusion, ready-to-fuse cells accumulate upstream of hemifusion. When using short-chain LPC, this inhibition is easily lifted by washing out this hemifusion inhibitor with fresh medium. Probing protein machines or cellular processes following release of trapped, synchronized cells (by adding calcium, providing contacts, or removing LPC) can be used to characterize the role these factors play during the membrane remodeling stage of cell fusions.

In addition to distinguishing the membrane remodeling stage of fusion from prefusion processes, it is also important to distinguish the fusion stage from the mechanisms that underpin the expansion of nascent fusion pores and fully unite the cytoplasms of fusing cells (*i.e.*, the ability of cells to exchange cytosolic markers versus fully integrating the organelles of multiple fusion partners into a multinucleated cell). These postfusion processes following a local merger of membranes are highly conserved in myoblast, osteoclast, and viral fusogen-mediated cell fusion and depend on the activity of dynamin, cell metabolism, PI(4,5)P_2_ concentration, and the actin cytoskeleton (21, 31, 32, 33). Note that the actin cytoskeleton and, specifically, finger-like protrusions of plasma membranes driven by multifilament actin structures bundled by dynamin also play an important role in priming the membranes for fusion (4, 34, 35).

In spite of the major challenges discussed above, recent studies have identified several proteins directly involved in the membrane remodeling stage of different cell–cell fusion processes, including: proteins of the Fusion Family in *C. elegans* development (EFF-1 and AFF-1) and gamete fusion (HAP2); myomaker and myomerger in myoblast fusion; FAST proteins in retroviral infection of nonenveloped viruses and syncytins in placentogenesis, osteoclastogenesis, and cancer cell fusion. Our readers can find in-depth discussions of these and other candidate fusion proteins, as well as the approaches used to identify them and characterize their contributions, in a number of recent papers (2, 3, 36, 37, 38, 39).

As our list of cell–cell fusogens and fusion-related proteins increases, we are continually faced with the shear complexity of the processes involved in forming the various multinucleated cell types discussed. Increasingly, it is becoming apparent that simply identifying protein fusion machinery is not enough, but we must also understand how cells manage these machines in a regulated manner toward the goal of eliciting the right cell fusions at the right time.

## Regulation of the membrane-remodeling stages of cell fusion

In addition to the proteins and protein complexes whose restructuring provides the energy required for membrane remodeling, cell fusions also depend on factors that activate this fusion machinery. The mechanisms that determine when cells begin to fuse remain elusive, but recent work has promoted several options for how cells might carry out fusion management. One option for regulating fusion is simply controlling the cell surface concentrations of protein fusogens. Indeed, many proteins implicated in cell fusion (*e.g.*, myomaker, myomerger, annexins in myoblasts and DC-STAMP, and annexins in preosteoclasts) are upregulated during prefusion differentiation (3, 21, 22). Even in the absence of changes in steady-state levels, cell surface concentrations of protein fusogens can be controlled by regulating their trafficking to and from the PM (40, 41). A second option for regulating cell fusions can be controlling the timing and efficiency of migration and adhesion processes, since fusion machinery is useless without appropriate membrane contacts. Finally, the timing of fusion can be controlled by the activation of protein fusogens already present at membrane contacts. Controlling mechanisms, where the fusogenic activity of protein machinery is unleashed by a specific triggering event, are characteristic for Ca^2+^-depended exocytosis (42), GTP hydrolysis-dependent fusion of endoplasmic reticulum membranes (43, 44), and fusion mediated by viral fusogens activated by low pH and/or proteolytic cleavage and/or receptor interactions and/or lipid cofactors (1, 30, 45). Each of these triggering mechanisms promotes the restructuring of protein fusogens and/or the recruitment of fusion factors to the site of fusion. Fusion dependence on specific triggers, rather than simply on the availability of protein fusogens, minimizes the risk of premature discharge of the conformational energy stored in fusion proteins, deters premature fusion/inactivation, and appears to be a consistent mechanism for regulating fusion processes. We still do not understand how cell fusions are regulated, but previous work seems to suggest that at least some are not regulated by controlling surface concentrations of fusogens. In the cases of trophoblasts and osteoclasts, the expression of the putative fusogen syncytin 1 and its receptor appears unchanged or reduced, when comparing naïve and fusion-committed cells (22, 46, 47).

While some of the proteins involved in rearrangements of lipid bilayers in fusion of diverse cell types have been identified, we still lack a complete picture of the mechanisms that regulate the activities of the fusion machinery that produces one cell from many.

### Phosphatidylserine exposure in nonapoptotic cells

Biological membranes differ considerably in their lipid compositions, which are tightly controlled by the cell, change dynamically, and demonstrate lateral heterogeneities (48). While the search is on for proteins that initiate and drive cell–cell fusion, the propensity of bilayers to form fusion intermediates also depends on membrane lipids. The presence of inverted-cone-shaped lipids (*e.g.*, LPC) and cone-shaped lipids (*e.g.*, diacylglycerol) strongly influences the energetics of hemifusion and fusion pores formed by bending the proximal and distal monolayers of the membranes, respectively (reviewed in (24, 49)). Because of the opposite net curvature of a lipid monolayer in a hemifusion connection (negative curvature favored by cone-shaped lipids) and in a fusion pore (positive curvature favored by inverted-cone-shaped lipids), the same lipid may have opposite effects on hemifusion and pore formation. For instance, while LPC in the proximal monolayers, as mentioned above, inhibits hemifusion, LPC in the inner leaflets promotes pore opening ((24, 50) but see (51)). The effects of monolayer-bending lipids on distinct stages of fusion are conserved between viral fusion, exocytosis, and cell–cell fusion (reviewed (2, 24, 49). In addition, some fusion processes depend on direct interactions between the proteins that mediate fusion and specific lipids, such as cholesterol, sphingomyelin, and ceramide (52, 53, 54). Moreover, many fusion processes depend on anionic lipids, including phosphoinositides, phosphatidic acid, bis(monoacylglycero)phosphate, and PS (30, 55, 56, 57).

An anionic aminophospholipid, PS only represents ∼4 mol % of the total phospholipids in cell membranes; however, it is almost exclusively retained in the cytofacial leaflet of the PM (*i.e.*, PS constitutes ∼20–30 mol % of the phospholipids the cytofacial leaflet versus 0–2 mol % in the exofacial leaflet) (58). PS exhibits cytofacial distribution throughout eukaryotic membranes by virtue of thermodynamic trapping, active transport, and low transbilayer mobility (58, 59, 60). Despite the tightly regulated asymmetry of PS distribution, many cells have the ability to transiently expose PS on the exofacial surface of the PM as a means of intercellular signaling. With its PM enrichment and the ability of many cells to trigger the exposure of PS on the exofacial surface of this membrane, it should come as no surprise that many cells appear to employ PS in the regulation of membrane fusion.

### Machinery that generates and releases transmembrane PS gradients

Eukaryotic PS asymmetry is largely owed to efficient sorting by P4-ATPases (also, flippases). P4-ATPases are transporters that selectively flip PS (and sometimes phosphatidylethanolamine) from the exofacial/luminal leaflet to the cytofacial leaflet (reviewed, (61)). This unidirectional transport is fueled by ATP hydrolysis, and Miraizumi and colleagues recently captured how ATP leads each step of this dance across the membrane at atomistic resolution (62). Estimates from model membranes suggest that, once oriented, PS should not spontaneously return to the exofacial/luminal leaflets on biologically relevant timescales (days) (63, 64). Loss of P4-ATPase function ablates PS asymmetry in eukaryotic membranes and leads to human disease (65, 66, 67, 68).

Much excitement over the last decades has revolved around PS when it is away from its cytofacial home visiting the exofacial surface of the cell. Thanks to all the molecular players and physical processes that retain PS on the cytofacial leaflet, controlled translocation of PS to the exofacial, PM surface can be used as an important signal in an ever-growing list of biological processes (reviewed, (69)). In many cases, externalized PS seems to coordinate important intercellular processes (*e.g.*, apoptotic engulfment, synaptic pruning, immune cell activation, etc.) (69, 70, 71, 72, 73).

Rapid exposure of PS on the cell surface as a means of membrane signaling was first described in the early 1980s and has continued to fascinate for decades (74). Excellent work by dozens of laboratories has elucidated several families of “professional” scramblases that facilitate membrane leaflet mixing in at least three independent pathways: Ca^2+^-activated phospholipid scrambling (Ca^2+^-PLS), caspase-activated phospholipid scrambling (Cas-PLS), and constitutive phospholipid scrambling (reviewed, (72, 75, 76)) (Table 1). We now recognize the role of at least one family of proteins that manages each of these scrambling pathways, although many questions remain concerning their functions in the diverse biological processes where cells employ exofacial PS signaling.

**Table 1:** Protein machinery that controls PS distribution across cell membranes and recognizes PS at the cell surface

PS organizing machinery	Putative functions	Role in fusion processes
TMEM16s C, D, F, E, G, J, and K	Nonselective, Ca^2+^-activated, bidirectional lipid scramblases of the PM and intracellular membranes (72, 192)	Myoblasts (141), osteoclasts (22), trophoblasts (149)
XKRs 4, 8, and 9	Elicit nonselective, caspase-activated, bidirectional scrambling at PM (98, 99)	?
Rhodopsin	Nonselective, constitutive, bidirectional scramblase of rod outer segments (193)	?
Adenosine and β-adrenergic receptors	Nonselective, constitutive, bidirectional scramblases (193)	?
P4-ATPases of the ATP 8, 9, 10, and 11 families	Flip PS from the exofacial to the cytofacial leaflet of the PM (61)	Myoblasts (169)
ABC transporter CED-7	Elicits PS exposure on axon PMs in *C. elegans* (194)	Axon regeneration (127)
PLSCRs	May play some role in eliciting PS exposure (79, 81)	Viral entry (82)

PS recognizing machinery		

Annexins	Soluble, PS-binding proteins that function as assembly factors in many biological processes (195)	Myoblasts (21, 196), trophoblasts (167), osteoclasts (22)
Lactadherin	Soluble, PS-binding protein (197)	Sperm-egg (168)
Protein S	Soluble, PS-binding protein (198)	?
GAS-6	Soluble, PS-binding protein (177)	Viral entry (199)
CD300 receptors	Membrane-bound receptors with affinity for exofacial lipids, some specifically bind PS (200)	Viral entry (201)
TIM receptors 1 and 4	Membrane-bound PS receptors with major roles in immunity (202)	Viral entry (203)
BAI receptors 1 and 3	Membrane-bound PS receptors (204)	Myoblasts (162)
Stabilin 2	Membrane-bound PS receptor (205)	Myoblasts (161, 163)
CD36	Membrane-bound PS receptor (206)	Macrophages (143)
PIEZO1	Mechanosensitive Ca^2+^ channel inhibited by exofacial, PM PS (207)	Myoblasts (169)
ADAM10 and 17	Metalloproteinase activated by exofacial, PM PS (208)	Sperm-egg, Myoblasts (209, 210)

It is important to note that scrambling PM lipids exposes many anionic lipids to the exofacial surface, not only PS. The role of these other anionic lipids in the effects linked to PS externalization remains to be explored. Moreover, the limited specificity of widely used PS probes (fluorescent annexin A5, lactadherin, and PS antibodies) also complicates the descriptions of PS exposure in the literature (reviewed, (77, 78)). PS-binding proteins drastically differ in their affinities and the dependence of these affinities on different factors. For instance, while cooperative Ca^2+^-dependent PS binding of annexin A5 has higher affinity (K_d_ as low as 0.1 nM versus 2–4 nM for lactadherin and TIM family receptors), calcium-independent binding of lactadherin is more sensitive and requires less than 2 mol % of PS versus more than 4 mol% of PS required for annexin A5 binding. Furthermore, PS binding by different proteins depends differently on membrane curvature, with lactadherin preferentially binding to highly curved membranes. Additionally, we still do not know whether the effects ascribed to PS exposure depend on its polar head group or only on its negative charge. Still, since PS content in PM is 10–30 times higher than the concentration of other anionic lipids (58), we are reasonably confident in ascribing the cell surface binding of annexins and PS receptors predominantly to externalized PS. In the future, it will be important to combine the different approaches currently used to assess PS exposure with analyzing the effects of masking specific cell surface lipids and rescuing perturbed PS exposure using application of specific exogenous lipids.

The search for the machines that elicit phospholipid scramblase (PLSase) activity has a long history. Early studies identified a protein, phospholipid scramblase 1 (PLSCR1), thought to catalyze Ca^2+^-induced movement of phospholipids between the inner and outer leaflet of the plasma membrane of erythrocytes in a bidirectional and nonspecific manner (79, 80, 81). Members of PLSCR family are expressed in multiple cell types and have been implicated in diverse processes both in healthy cells and in many diseases, including viral infections (81, 82). Early reconstitution of the scrambling activity of PLSCR in liposomes (81) suggested that this protein may directly mediate lipid redistribution between membrane leaflets; however, the timescales of scrambling observed in these experiments were nearly 20 times slower than the scrambling observed in native membranes. Moreover, normal levels of Ca^2+^-induced phospholipid scrambling are observed in mice deficient in PLSCR (81), in cells with suppressed PLSCR (83) and in flies lacking both of the PLSCR homologs in *Drosophila* (84). While these and other findings argue against the essential role of PLSCR as a PLSase (reviewed in (69, 81), it remains possible that PLSCR deficiencies can be functionally compensated for by other types of scramblases or that PLSCR functions as a component of a protein complex that retains some scrambling activity in the absence of PLSCR. It has been also suggested that the role of PLSCR-1 in PS redistribution is limited to specific signaling pathways such as PS exposure at the interphase between secretory granules and the PM during exocytosis in neuroendocrine cells (85).

TMEM16F is now recognized as a prototypical Ca^2+^-PLSase in platelets and a variety of other cell types (86). Many TMEM16 PLSases are recognized as both necessary and sufficient for Ca^2+^-PLS in a variety of cell types. Moreover, discreet TMEM16 domains are appreciated for their roles in ferrying lipids between leaflets, and atomistic structures have resolved many of the mechanistic steps TMEM16 PLSases undergo to disrupt lipid concentration gradients across bilayers (87, 88, 89, 90, 91, 92, 93, 94). Current understanding of the mechanism by which TMEM16 PLSases facilitate transbilayer lipid movement closely resembles the elegant “credit card” model suggested by Pomorski and Menon (95). When activated by Ca^2+^, the transmembrane helices of a TMEM16 PLSase create a hydrophilic cleft through the membrane core. Once this hydrophilic pathway is opened, lipid head groups are provided with a leaflet-to-leaflet bridge while their oily tails remain in the membrane’s hydrophobic core. Once this bridge is created, lipids simply run down their concentration gradients, previously created by transporters, in a nonselective manner (94, 96, 97).

In contrast, to TMEM16s the XKR protein family elicits Cas-PLS activity essential for PS exposure during apoptosis and the engulfment of apoptotic cells (98, 99). Although both Ca^2+^-dependent and caspase-dependent pathways lead to PS exposure, these scrambling pathways are functionally separate, permitting one to operate independent of the other (98, 100). Caspase activity leads to the cleavage of XKR proteins and nonspecific scrambling of PM lipids, including PS (98, 99). Recent reports also demonstrate that XKR proteins can be activated by phosphorylation, independent of caspase cleavage, and elicit PS exposure, although the biological consequence of this route of activation remains to be elucidated (101). XKR-mediated Cas-PLS is considerably less characterized in comparison with TMEM16-activated Ca^2+^-PLS, and many questions concerning the mechanism of XKR-elicited PS exposure remain (*e.g.*, do XKR proteins themselves facilitate the exchange of lipids between leaflets or are other complex members required?). Moreover, the lack of structural information concerning this protein family limits our understanding of how changes in XKR protein structure relate to its function in Cas-PLS.

Although both Ca^2+^-PLS and Cas-PLS elicit PS exposure, considerable differences exist between the PS signal these pathways produce including: the timing between activation of the pathways and PS exposure, the spatial organization of the PS exposed, and the reversibility of PS exposure. To begin, PS exposure *via* Ca^2+^-PLS has been observed on the order of 5–15 min after stimulation compared to >1 h between apoptotic stimulation and observed PS exposure in Cas-PLS (98). Although, this difference likely reflects the speed of Ca^2+^ release versus the stepwise activation of the caspase cascade and subsequent cleavage of substrates. These two signaling processes also exhibit characteristic differences in the distribution of cell surface PS on the PM. Nonapoptotic PS exposure associated with Ca^2+^-PLS produces discrete, punctate regions of PS probe binding at the PM (22, 30, 102, 103, 104, 105, 106, 107) compared with the stronger, uniform PS signal observed on the PM of apoptotic cells. How exofacial PS would form discreet patches remains an open question. Externalized PS can predominately partition into particular PM domains, such as cholesterol-enriched rafts (108). Indeed, both PS and TMEM16F are enriched in lipid rafts (109, 110, 111). PS-enriched puncta can also be formed and stabilized by cooperative binding of PS-binding proteins, such as annexin A5 (112). Finally, PS domains could be the result of local activation of Ca^2+^-PLSases and deactivation of P4-ATPases. Spatially restricted Ca^2+^ signaling could accomplish both the activation and deactivation mentioned, and as PS diffused outside of this domain, it would be returned to cytofacial leaflet by transporters outside the elevated Ca^2+^ zone. Spatially restricted Ca^2+^ signaling activates a close relative of the TMEM16 scramblases, the Ca^2+^-activated Cl^-^ channel TMEM16 A, in this manner (113). In contrast, Cas-PLS associated with apoptosis is typically observed across the entire PM. Mechanisms that underlie Ca^2+^-PLS -dependent generation of PS-enriched membrane domains in the outer leaflet of the cell, the lifetimes of these domains, local PS concentrations, and the functional importance of heterogeneity in PS distribution at the PM remain to be determined. In addition, it appears that the reversibility of the PS exposure elicited by Ca^2+^-PLS versus Cas-PLS likely differs, if for no other reason than by how each pathway inhibits P4-ATPases from obscuring PS exposure. P4-ATPases are inhibited during Ca^2+^-PLS by Ca^2+^ binding, either directly or indirectly, and this process is reversible (114, 115, 116). In contrast, P4-ATPases are cleaved by caspases, rendering them nonfunctional during Cas-PLS (117). In the absence of subsequent inhibition of caspase activity and expression/trafficking of new transporters, Cas-PLS is likely nonreversible if initiated on a PM-wide scale. By nature of their respective signaling pathways’ effects on P4-ATPases, Ca^2+^-PLS maintains the ability to function as a transient intercellular signal, whereas caspase dependence renders exofacial PS delivered *via* Cas-PLS an enduring signal.

**Figure 2:**
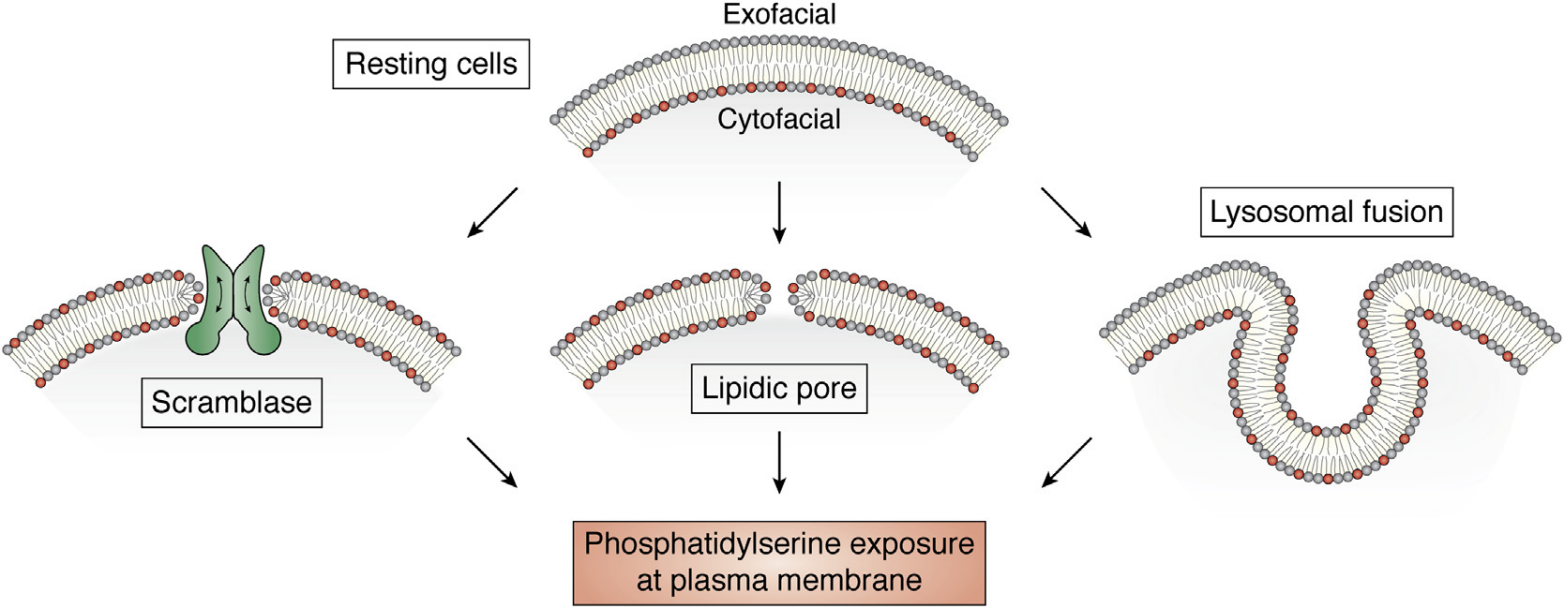
**Some mechanisms of phosphatidylserine (PS) exposure at the plasma membrane in nonapoptotic cells.** Cartoon depicting potential mechanisms of PS exposure in biological processes. At rest, PS (shown as lipids with *red polar heads*) is asymmetrically oriented toward the cytosolic face of the plasma membrane (*top*), however in some biological contexts PS is transiently exposed on the extracellular facing leaflet by the activation of professional phospholipid scramblases (*left*, *middle*), the formation of lipidic or partially lipidic pores (*center, middle*), or fusion between PM and intracellular vesicles carrying PS in their inner (lumen-exposed) leaflet (*right, middle*).

While many examples of PS exposure are known to involve the professional scramblases discussed above, in some cases exofacial PS exposure may be facilitated by other mechanisms (Fig. 2). Since PS is concentrated in the luminal leaflet of some of the intracellular membrane organelles, PS-enriched patches can mark the sites of the fusion of these organelles to PM. Indeed, PS clusters mark the sites of lysosome–PM fusion (118). The exocytotic fusion sites are also marked by the appearance of exofacial PS (119, 120). Note, however, that PS exposure at the exocytotic fusion sites can involve the activity of PLSCR-1 (85) at either the PM or the granule membrane. Besides PM-fusing vesicles, PS-enriched patches can represent the budding of extracellular vesicles (108, 121). Merger of exofacial and cytofacial leaflets of the PM by the opening of lipidic or partially lipidic pores induced by electric fields, insertion of peptides, or insertion of nucleic acid can also promote lipid scrambling and PS exposure (122, 123, 124, 125, 126). Additionally, we still do not know the mechanism of PS exposure in a number of biological processes including the accumulation of PS on injured axons requiring the ABC transporter CED-7 (127), as well as the reversible PS exposure induced by galectin 1 (105) or by introduction of the viral proteins HIV-GAG or Ebola virus VP40 (128).

### Fusion-unrelated processes dependent on PS signaling

Transient PS exposure was first recognized as a highly specialized signal in platelets and erythrocytes, but subsequent implication of exofacial PS in the recognition and removal of apoptotic cells expanded interest in PS signaling as a more general phenomenon (74, 129). Exofacial PS in activated platelets provides a platform for the assembly line of clotting factors that convert prothrombin to thrombin, which facilitates the formation of a fibrin-based, secondary hemostatic plug at sites of injury (130). PS and a number of PS receptors function in the recognition and phagocytic removal of apoptotic cells (131). Although the role of PS in apoptosis led to biology courses ubiquitously referring to PS as an “eat me” signal, exofacial PS is necessary but not sufficient to elicit phagocytosis (132). Both coagulation and apoptosis are associated with the significant release of extracellular vesicles decorated with exofacial PS (133, 134). Exofacial PS and the PLSase machinery that exposes it are implicated in the production of these and other flavors of vesicular signaling bodies (121, 135). PS-exposing extracellular vesicles are widely considered important messengers mediating intercellular communications (136). Exposed PS is required for proper antigen recognition in B-cells (103) and T-cells (137), the protection of T-cells from their own pore-forming protein used to degrade target cells and the Ca^2+^-PLSase TMEM16F plays an essential role in the recognition and clearance of bacteria in the innate immune response (138). Moreover, proper assembly of our neuronal circuitry owes its tuning to PS signaling in synaptic pruning (139). These are just a few examples of the role exofacial PS plays as a common cell–cell signaling module often associated with coordinating intercellular functions toward a common goal in tissues.

In short, PS is a cytofacial PM phospholipid whose exofacial exposure is utilized by cells to coordinate a number of highly orchestrated intercellular processes, from clotting to synaptic pruning. Proteins that organize the orientation of PM PS function as signaling machinery in an ever-growing list of biological processes.

## Phosphatidylserine signaling as a uniquely conserved signaling module in cell fusion

### PS exposure in cell–cell fusion

A link between cell-fusion processes and exofacial PS exposure was first described for the formation of multinucleated skeletal muscle fibers (102), but to our knowledge, PS exposure is now associated with all cell-fusion events where this signaling process has been seriously evaluated (Fig. 1). Exposed PS was found to accompany the formation of multinucleated skeletal muscle fibers and myoblast fusion without apoptotic changes or dependence on caspase activity (102, 140). Appearance of PS at the surface of fusion-committed myoblasts generally correlated with the timing of cell–cell fusion events, with no PS found at the surface of proliferating cells and mature myotubes (102). Suppressing the expression of the Ca^2+^-PLSase TMEM16E inhibits myoblast fusion associated with muscle repair (141). Intriguingly, the Cas-PLSase XKR8 also plays a role in myogenic commitment of myoblasts; however, whether the role of XKR8 in myogenesis involves PS exposure is not clear (142).

Macrophage fusion to form multinucleated giant cells also involves PS. These cell fusions are accompanied by PS exposure, inhibited by blocking cell surface PS, and require the PS receptor CD36 (10, 143). Formation of multinucleated osteoclasts by the fusion of osteoclast precursors also involves cell surface exposure of PS as well as another aminophospholipid normally residing in the inner leaflet of the PM, phosphatidylethanolamine (144). Both inhibiting PS exposure by suppressing the activity of TMEM16 scramblases and blocking PS at the cells’ surface inhibit osteoclast fusion (22).

Fusion of trophoblasts in placentogenesis has been also linked to PS exposure. The fusion of several carcinoma models of human trophoblast fusion involves PS exposure (*e.g.*, BeWo cells treated with forskolin) (145, 146, 147). Moreover, anti-PS antibodies have been associated with abnormal placental development and adverse pregnancy outcomes (148), suggesting that exposed PS plays a role in human placental development. Additional evidence for the importance of PS signaling in trophoblast fusion comes from a recent paper showing the dependence of BeWo cell fusion and murine placentogenesis on the Ca^2+^-PLSase TMEM16F (149).

Not all cell fusions linked to PS exposure produce multinucleated syncytia. Fertilization is also associated with PS exposure. Following introduction to the female reproductive tract, sperm undergoes capacitation and the acrosome reaction, whereby the sperm head undergoes a series of membrane rearrangements in preparation to penetrate the zona pellucida, adhere to the egg, and undergo sperm–egg fusion (150). These prefertilization steps result in PS exposure on the head of sperm that is required for efficient fusion with the oocyte (73).

The link between PS exposure and cell fusion is not limited to the cells destined to fuse in normal physiology. Cancer cells, whose fusion is thought to contribute to the progression of cancer (11), often display PS at their surface (151). Cell repair elicited by PM insults also employs exofacial PS. Exposure of PS on injured axons initiates axonal fusion, a highly efficient mechanism of nervous system repair (reviewed, (152, 153)). This finding is especially interesting because this fusion process involves the *C. elegans* fusogen EFF-1, and to the best of our knowledge, this is the only indication in the literature suggesting that fusions mediated by “Fusion Family” (FF) proteins (including *C. elegans* EFF-1 and AFF-1 and gamete-expressed HAP2) may, as all other fusion processes discussed above, depend on PS exposure. Other “self fusion” events beyond axonal fusion also have links to PS. Efficient PM patch repair following physical insult or exposure to pore forming toxins involves a number of PS-binding proteins (*e.g.*, MG53/TRIM72; Annexins 4, 5, and 6; Dysferlin) (154, 155, 156, 157) and Ca^2+^-PLSases (TMEM16 E and F) (158, 159). TMEM16F-dependent PM scrambling, associated with PS exposure, appears critical for the efficient repair of PM damage elicited by pore-forming toxins (159). Moreover, dysferlin-dependent accumulation of exofacial PS at the site of membrane lesions is crucial for the removal of damaged PM patches *via* macrophages (156).

In spite of the many lines of evidence supporting the association between PS externalization and cell–cell fusion, the role and the place of PS signaling in the fusion of PMs remain poorly understood.

### Interpreting a PS signal from a fusion partner

PS is exposed between fusing cells in numerous systems as an intercellular signal, but what is a signal without the ability to interpret it? Recent advances have begun to shed light on some of the relay machinery cells use to interpret and transform PS signaling into a biologically meaningful message between fusion partners (Table 1). PS receptors and other PS-binding proteins play a major role in the recognition and clearance of apoptotic cells (*e.g.*, Tim4, BAI1, stabilin 2 (STAB2), annexin A5, and other annexins) (160). Similarly, PS receptors STAB2 and BAI1 are recognized for their roles in interpreting PS signals in myoblast fusion. STAB2 promotes efficient fusion of myoblasts *in vitro* and fusion-dependent muscle regeneration *in vivo* (161). In this study, application of exogenous PS was found to promote fusion for wild-type myoblasts but had no effect on fusion of STAB2-deficient myoblasts, emphasizing the role of STAB2–PS interactions in the STAB2 dependence of myoblast fusion.

PS receptors BAI1 and BAI3 have both been recognized for their roles in regulating myoblast fusion by detecting PS exposure and eliciting fusion between partners *via* the ELMO/Dock180/Rac1 signaling pathway (162, 163). Remodeling of the actin cytoskeleton in this pathway appears to be an at least partially shared requirement for syncytial formation during myoblast and macrophage fusion (164). However, a recent report demonstrates that BAI1 is nearly undetectable in fusing muscle cells, making its previous link to myoblast fusion difficult to interpret (161). Similarly, the PS receptors BAI1, BIA3, and CD36 were all found to play major roles in the fusion of sperm and oocytes through the ELMO/Rac1 signaling pathway. The shared PS recognition machinery between fertilization and myoblast fusion leads to the surprising discovery that sperm can fuse with skeletal muscle in a PS-dependent manner (73).

The list of protein players shared by different cell fusions also includes CD36 and annexins. The PS receptor CD36 facilitates myoblast fusion and macrophage fusion (143, 165). Development of fusion competence for both myoblasts and osteoclast precursors is associated with a strong increase in the amount of the annexins A1 and A5 present at the cell surface and depends on these exofacial, PS-bound annexins (21, 22). Trophoblast fusion also depends on annexin A5. Based on anti-annexin A5 antibody binding, at the time of most intensive syncytium formation, the surface of the trophoblasts is covered with endogenous annexin A5 (166). The importance of annexin A5 in trophoblast fusion has been also documented by Degrelle *et al.* (167); however, in this case the authors suggest that annexins participate in fusion by binding to the inner leaflet of PM of trophoblasts. Prostate cancer cell fusion also depends on annexin A5, and its expression is increased in prostate cancer tissues compared with nonmalignant tissues (13).

Beyond receptors and annexins, some other PS-recognizing proteins also prove vital for cell fusions. The PS-binding protein lactadherin is released into the female reproductive tract along with sperm, binds PS exposed on the sperm head, and is required for sperm to bind the egg zona pellucida, suggesting lactadherin may function as a PS-binding protein in fertilization (168). Additionally, the mechanosensitive Ca^2+^ channel PIEZO1, acting as a molecular break halting myoblast fusion, is inhibited by PS exposure (169).

Slowly, the machinery required to interpret PS signaling between fusion partners is being resolved; yet, identification of the machinery is the first step in elucidating the mechanistic role PS exposure plays in cell fusion. Of note, cell fusion’s dependence on PS-binding proteins also argues against the hypothesis that PS exposure at contacting leaflets of the apposed PMs is sufficient to facilitate calcium-induced, protein-independent fusion by mechanisms described for protein-free lipid vesicles composed of PS (see below).

The involvement of PS-binding proteins in many cell-fusion processes further substantiates the functional importance of PS exposure in diverse cell fusion processes. Can cell fusion proceed without PS externalization and PS-binding proteins? To the best of our knowledge, the absolute requirement for PS in the cell-fusion process has never been reported. In most cases, treatments targeting appearance or accessibility of exofacial PS or PS-binding proteins inhibit, sometimes strongly inhibit, but not abolish fusion. Possibly, after these treatments, the residual levels of accessible exofacial PS and bound PS receptors remain high enough to support background levels of fusion. Alternatively, there may be levels of redundancy built into cell–cell fusion that bypass the need of PS as a regulatory signal to some extent, as is the case in exocytosis, where low levels of fusion are observed even in the presence of high concentrations of cytosolic chelator (170).

### PS functions at the membrane remodeling stage of cell fusion

Recent studies indicate that PS exposure is required for postdifferentiation fusion processes, specifically, PM remodeling. Loss of the PS receptors BAI1 and STAB2 greatly diminishes syncytia formation but does not inhibit early myogenic differentiation (161, 162). Suppressing expression or activity of PS-binding annexins A1 and A5 inhibits membrane remodeling stage of myoblast fusion decoupled from prefusion processes by LPC synchronization (21). These data indicate that extracellular annexins and, by extension, cell surface PS regulate muscle cell fusion downstream of the prefusion differentiation processes. It remains possible that, in addition to their role in membrane fusion, PLSases, PS exposure, and PS-binding proteins also contribute to prefusion stages. To this point, suppression of the Cas-PLSase XKR8 appears to hamper early stages of myogenic differentiation (142). Note, however, that Xkr8’s role in myoblast differentiation is not related to PS externalization.

Similar to skeletal muscle fusion, we find that osteoclast fusion decoupled from prefusion processes by LPC synchronization depends on PS externalization (22). Inhibiting TMEM16 PLSases, masking exofacial PS, or perturbing the expression or activity of the PS-binding proteins annexins A1 or A5 greatly impeded fusion between osteoclast precursors. These experiments strongly suggest that the role of PS exposure in this system lies in the membrane remodeling stages of cell fusion rather than in some upstream steps of differentiation.

At this point we would like to note that despite many studies supporting the role of PS in cell fusion, to the best of our knowledge, the field of PS signaling still lacks experimental approaches allowing unambiguous demonstration that cell fusion or other cell biological processes depend on PS exposure rather than some other lipids delivered to the outer leaflet by the loss of membrane asymmetry. To deal with this technical limitation, it is important to combine different approaches to robustly support the specific role for PS exposure in biological processes being evaluated, including: targeting the expression and function of scramblases, targeting PS receptors and PS-binding proteins; analyzing the effects of masking specific cell surface lipids; and evaluating the application of specific, exogenous lipids. A particularly useful example of employing contrasting approaches to evaluate the requirement of exposed PS is myoblast fusion. Several recent papers have demonstrated that myoblast fusion is inhibited by removing scramblase activity (141), by reducing or removing PS-binding annexins (21), by removing the receptor STAB2 (161), or by masking PS (161), and myoblast fusion is promoted by exogenous PS (161).

As an additional note, redistribution of PS from the inner to the outer leaflet of the PM also necessarily decreases the PS content of the cytofacial leaflet of the PM that regulates the localization and function of many intracellular, cationic proteins (171). While these effects likely influence cell–cell fusion processes, the vital role of exofacial PS is evidenced by the ability to inhibit diverse types of cell–cell fusions by masking exofacial PS and by fusion’s dependence on PS receptors and extracellular PS-binding proteins in these processes.

### PS regulation in intercellular and viral fusion

Since the precise role of PS in cell fusion is still coming into focus, perhaps we can learn from much better characterized examples of PS-dependent fusion processes. The ability of PS to support membrane fusion was first reported almost 50 years ago in the pioneering studies of Papahadjopoulos and co-workers, in the fusion of liposomes—protein-free vesicles—where PS is required for Ca^2+^-induced fusion (172).

PS also regulates membrane fusion in exocytosis. Raising PS content in cell membranes promotes the opening of fusion pores and increases their lifetimes in SNARE-dependent, Ca^2+^-triggered intracellular fusion (42, 173). To this point, suppressing PS delivery to the inner leaflet of the PM or the cytosolic leaflet of exocytotic vesicles inhibits insulin secretion (174).

The membrane fusion required for entry of HIV, Zika, Ebola, and many other enveloped viruses into their host cells depends on PS in the viral envelope (175), on scramblases such as Xkr8, which delivers PS to the outer leaflet of Ebola (176), and on PS receptors on the surface of host cells (177, 178). Entry of some viruses also depends on PS exposure in the outer leaflet of host PMs (179). For example, the binding of alphaherpesvirus glycoprotein H to α4β1 host integrins activates Ca^2+^ signaling and induces PS exposure on the host cell PM (107). Blocking either Ca^2+^ or PS signaling inhibits alphaherpesvirus infection. Similarly, in the case of HIV-1 entry, interactions between gp120 and host CD4 receptors and G protein coreceptors induce Ca^2+^ signaling and trigger TMEM16F-mediated PS exposure that promotes fusion (30).

Studies of Ca^2+^-mediated liposome, intracellular, and viral fusion processes have suggested a number of distinct mechanisms of fusion regulation by PS. PS-enriched protein-free bilayers were found to establish very tight, dehydrated, or partially dehydrated contacts facilitating formation of fusion intermediates (180). In synaptic exocytosis, negatively charged PS promotes membrane recruitment and oligomerization of the primary Ca^2+^ sensor synaptotagmin and penetration of its PS-bound polybasic lysine patch into the membrane (42, 181, 182). Similarly, PS–synaptotagmin interactions have also been suggested to accelerate vesicle fission in clathrin-mediated endocytosis (183). Finally, PS on target cell membranes has been suggested to draw out positively charged regions of the fusogens of dengue, vesicular stomatitis viruses, and HIV to facilitate their fusogenic restructuring and oligomerization (30, 57, 184, 185).

In brief, PS can promote membrane fusion events through a number of distinct mechanisms, including direct effects on the fusogenic properties of lipid bilayers, recruitment of fusion machinery components, and by facilitating the restructuring of fusion proteins.

### PS exposure as a conserved regulator of assembly and activation of cell-fusion machinery

As discussed above, exposed PS functions as an essential and strikingly conserved cell–cell signal required for syncytium formation in many important cell-fusion processes. Partners interpret this oily, anionic signal as instructions for fusion, so much so that PS-exposing sperm fuses with muscle cells in a PS-dependent manner (73). Not only is exofacial PS required for myoblast fusion, but its return to the inner leaflet of PM has been suggested to act as a fusion brake in a mechanism, whereby the inward translocation of PS inhibits excessive fusion by activation of PIEZO1 (169). As illustrated above, our understanding of the many intricacies of cell fusion events and their links to PS is still incomplete. For instance, we still do not know whether cell fusions in *C. elegans* mediated by EFF-1 or AFF-1; and gamete fusion mediated by HAP2 are associated with PS exposure or the precise mechanisms by which PS influences the fusion of mammalian cells. However, the many lines of evidence discussed above and a loose analogy to the trigger-dependent viral and intracellular fusions bring us to a final unifying hypothesis: PS exposure acts as a conserved trigger for the membrane remodeling phase of cell fusion.

The mechanisms that initiate and control prefusion externalization of PS in cell–cell fusion processes remain largely unexplored. We suggest that intracellular Ca^2+^ signaling activates Ca^2+^-PLS and mediates PS exposure in coordination with the late stages of prefusion processes, such as the formation of prefusion cell–cell adhesion structures or invadosomes. Indeed, myoblast fusion (186), osteoclast fusion (187), and trophoblast fusion (188) depend on cadherin cell–cell adhesion molecules and cadherin-mediated cell–cell contacts that trigger Ca^2+^ influx and Ca^2+^ release from intracellular stores. These contacts generate increases in intracellular Ca^2+^ in the vicinity of intercellular junctions (189). Self-contact-induced membrane fusion that eliminates the contacts between different regions of the same PM in epithelial cells also depends on cadherins (190).

**Figure 3:**
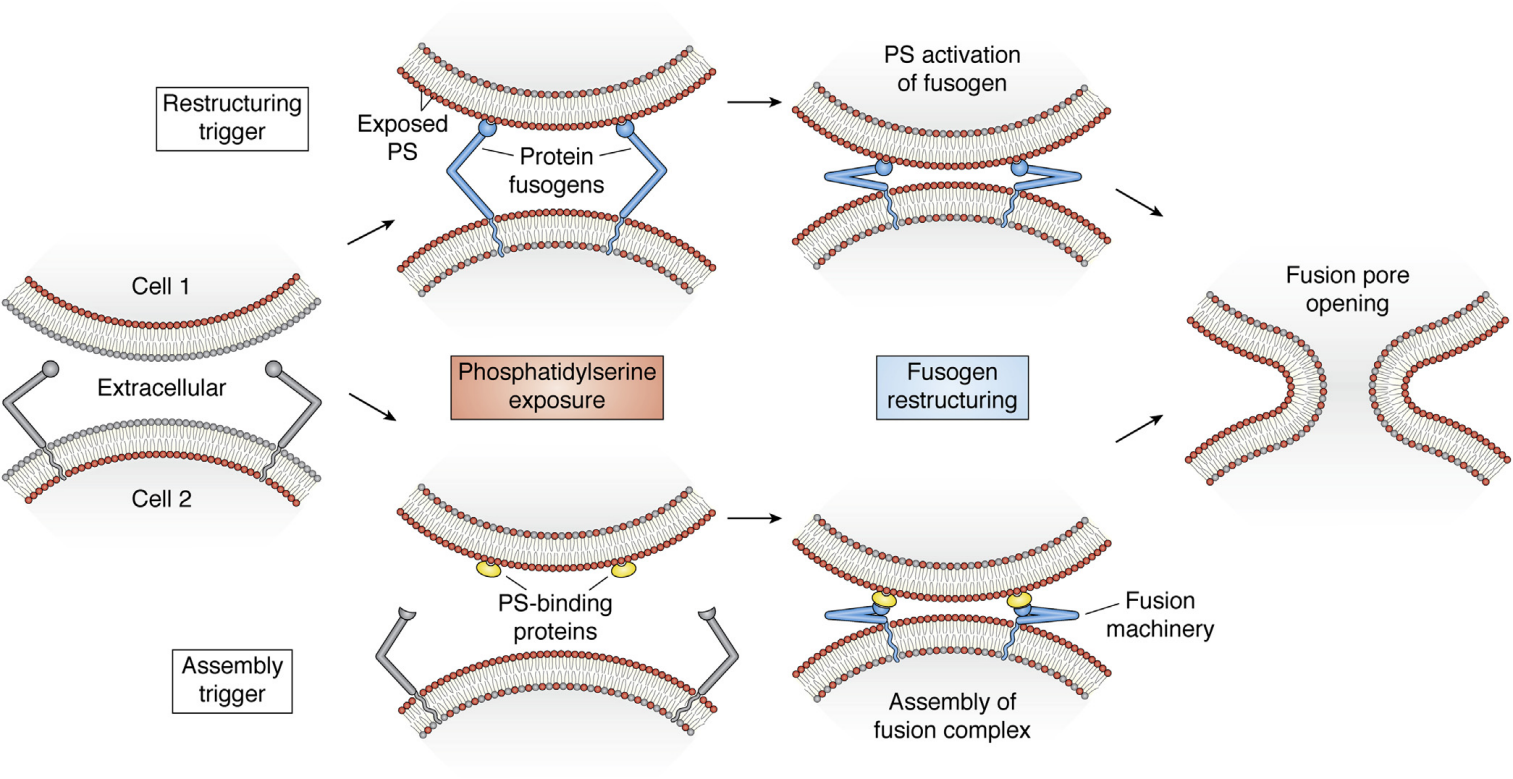
**Proposed mechanisms of cell-fusion regulation by PS exposure.** We suggest that exofacial PS either directly promotes fusogenic restructuring of protein fusogen (illustrated by the pathway at the *top*) or triggers assembly of the fusion machinery (pathway at the *bottom*). Starting from the *left*, two fusion-committed cells prepare membrane fusion. In the first scenario, binding of protein fusogens (*blue*) to PS (*red*) exposed at the surface of the cells triggers restructuring of the fusion, depicted here by the hypothetical closing of a “hinge-like” fusogen. In the second scenario, PS interactions with PS-binding proteins (*yellow*) trigger assembly and activation of the fusion machinery. Finally, the conformational energy discharged in either mechanism promotes the union of fusion partner membranes and the opening of a fusion pore between them (*right*).

Specific mechanisms that underlie PS dependence during the membrane remodeling stage of diverse cell–cell fusion processes also remain to be resolved. We suggest that this mechanism may take one of two forms: exofacial PS either triggers fusogenic restructuring of protein fusogens or directs the assembly of protein machinery at the site and time of fusion (Fig. 3). The first of these scenarios is related to the mechanism discussed above by which PS in host, target membranes promotes HIV Env-mediated fusion. Syncytins, endogenous retroviral envelope proteins involved in trophoblast fusion, osteoclast fusion, and cancer cell fusion (reviewed, (2)), share many essential structural elements with HIV Env. Therefore, exofacial PS may trigger syncytin-mediated fusion through a mechanism analogous to the PS-dependent activation of HIV Env-mediated fusion. In this proposed mechanism, exofacial PS promotes fusogenic restructuring of proteins that are already present at the fusion site. In an alternative mechanism analogous to the suggested role of PS in SNARE-mediated fusion, PS interactions with PS-binding proteins may direct the timing and location of fusion protein assembly. In this scenario, the patchy, exofacial PS associated with nonapoptotic scrambling may function to organize fusion machinery similar to how the blinking lights of a runway direct air traffic. Discreet PS domains facilitate the recruitment and arrangement of fusion complex components at future fusion sites and communicate this preparedness to the cell’s fusion partners. Indeed, cell-surface-associated, PS-binding annexins are known to directly bind and assemble many proteins found to promote fusion (such as S100 proteins in osteoclast fusion) (22). Moreover, the assembly of PS-binding proteins may promote bending in the membranes where they are recruited. These deformations could generate early fusion intermediates and/or promote fusion by creating complexes that bring membranes into closer apposition, as suggested for synaptotagmin (173, 191) and for osteoclast fusion (22). In analogy to the triggers that elicit intracellular and viral fusion, we propose that exofacial PS in cell–cell fusion functions as a “Fuse Me” signal. Even as we lack a complete understanding of the mechanism(s) by which PS regulates membrane remodeling, a wealth of recent findings strongly support that PS exposure controls the efficiency and timing of cell fusion.

## Concluding remarks

Diverse cell–cell fusion processes involve different types of cells, develop in varied biological contexts, and are preceded by unique differentiation processes. Intriguingly, the membrane remodeling stage of these diverse cell-fusion processes is preceded by and depends on the nonapoptotic exposure of PS at the surface of fusion-committed cells. Cell surface PS is widely considered one of the apoptotic “eat-me” signals. Studies on regeneration of transected axons and PM repair indicate that PS exposure also acts as a “save-me” signal that triggers segment fusion/reconnection, maintaining axonal integrity, and PM resealing. Here, based on the abundant evidence supporting the role of PS exposure in diverse cell-fusion events, we propose that PS also acts as a “fuse-me” signal. If exofacial PS in effect identifies ready-to-fuse cells, PS could be used as a target and/or a biomarker for the development of novel therapies targeting cell-fusion processes associated with human disease. Understanding the molecular mechanisms of cell–cell fusion processes in health and disease will require not only identification of the components of the fusion machinery but also dissection of both conserved and process-specific pathways underlying the externalization of PS and the PS-dependent assembly and/or function of proteins that mediate cell–cell fusion.

## Conflicts of interest

The authors declare that they have no conflicts of interest with the contents of this article.
